# Corrosion of Porous Building Ceramics Caused by Double Sulphate Salt

**DOI:** 10.3390/ma18051041

**Published:** 2025-02-26

**Authors:** Wojciech Wons, Ewelina Kłosek-Wawrzyn, Karol Rzepa

**Affiliations:** Department of Building Materials Technology, Faculty of Materials Science and Ceramics, AGH University of Krakow, A. Mickiewicza Av. 30, 30-059 Krakow, Poland; wwons@agh.edu.pl (W.W.); krzepa@agh.edu.pl (K.R.)

**Keywords:** double salt corrosion, building ceramics, efflorescence

## Abstract

Porous materials are subjected to the corrosive effects of soluble salts. This corrosion, typically known as efflorescence, is primarily superficial. However, internal corrosion within the material is also frequently observed. This article presents a simulation of volumetric damage in sintered porous ceramic materials (made of clay (75 vol.%), quartz sand (10 vol.%), and sawdust (15 vol.%), fired at 950 °C), caused by the crystallization of double salts, specifically ploweite (6Na_2_SO_4_·7MgSO_4_·15H_2_O) and/or glauberite (CaSO_4_·Na_2_SO_4_). The exact mechanism responsible for the formation and interaction of these salts has yet to be fully comprehended. It is established that this mechanism occurs in ceramic materials containing calcium compounds and in mixtures of Na_2_SO_4_ and MgSO_4_ salts. Dissolved Na_2_SO_4_ acts as a substrate for the formation of glauberite, while dissolved MgSO_4_ participates in intermediate reactions (which are necessary for the creation of glauberite).

## 1. Introduction

**Figure 1:**
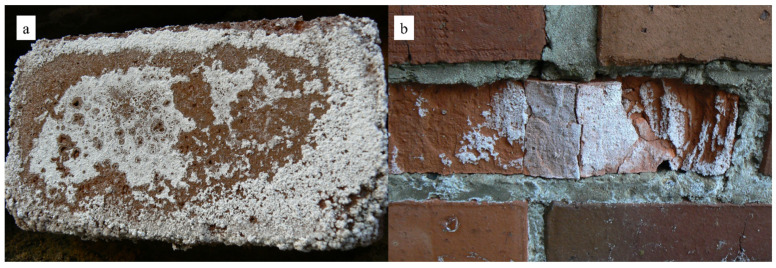
Surface (**a**) and volume damage (**b**) caused by soluble salts (reproduced with permission [9]).

Soluble salts are a prevalent cause of corrosion in porous building materials, including ceramics, concrete, cement mortars, and natural stone materials [1,2,3,4]. These salts cause efflorescence, as depicted in Figure 1a. In addition to reducing aesthetic value, they can also cause subsurface layers to flake off (referred to as sub-florescence). In some cases, they can cause cracks and damage to the bulk of the material, as shown in Figure 1b. Efflorescence can be attributed to salt crystallization (crystallization pressure) or volume changes during hydration (hydration pressure) [5,6,7]. The efflorescence mechanism is intricate, as it necessitates the simultaneous fulfillment of three conditions:

- Presence of soluble salt sources, whether internal (within the material) or external.
- The material must feature capillary pores.
- The material must be in direct contact with water [4,8].

Only when these conditions are satisfied can salt dissolved in water migrate through the material’s capillary pores, subsequently leading to salt crystallization triggered by evaporation and/or temperature fluctuations.

The effects of efflorescence are contingent upon several factors, encompassing the type of material, the corrosive salts involved, and prevailing humidity and thermal conditions. In their study on moisture and ion transport within fired-clay brick, Pel et al. [10] established that the extent of salt efflorescence is primarily governed by humidity and the rate of drying. Typically, materials with greater porosity are more susceptible to the adverse impacts of efflorescence. Consequently, concrete, characterized by low porosity, exhibits greater resistance to efflorescence in comparison to porous masonry ceramics [1,2]. This article presents salt corrosion of building ceramics; therefore, the following part focuses on the influence of soluble salts on these materials.

There are numerous methods for testing the effect of salt efflorescence/crystallization on building ceramics. S. Pietruszczak et al. [11] used a method based on cycles of saturating the samples with a corrosive solution and subsequent drying. The impact of salt is assessed by measuring the bulk density, porosity, and bending strength of the samples. M. Dubale et al. [12] used the method described in the Indian standard IS: 3495. A group of scientists working at The RILEM Technical Committee 271 ASC prepared a review report on testing methods for salt crystallization in porous building materials [13]. The report overviewed methods presented in the literature and focused on the differences regarding: the type of material samples tested; type and amount of used salt; method of saturating the sample with salt; drying conditions; and method for assessing damage caused by a corrosive factor. Based on the above report, scientists from RILEM Technical Committee 271-ASC proposed a method for testing the corrosion of porous building materials caused by salts based on drying and saturation cycles with sodium chloride or sodium sulphate salt [14,15]. The effect of the corrosive agent is assessed by visual observation and measurement of the sample’s mass loss. A. Parisotto et al. [16] used the colorimetric method to assess the impact of corrosion. Efflorescence quantity comparison was determined by the difference of color index measured by a dedicated device. V.A. Anupama and Manu Santhanam [17] used a method based on saturation/drying cycles modified by wind simulation during drying.

**Figure 2:**
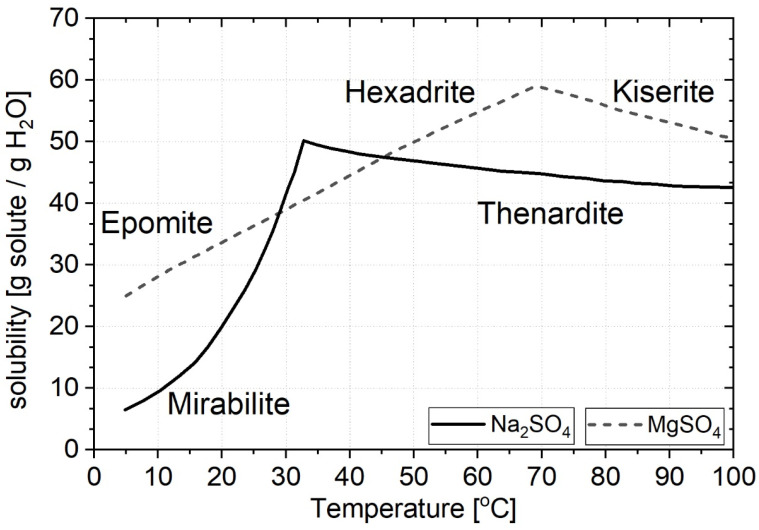
Solubility of salt; based on [29].

Sulphates and carbonates are the most encountered compounds in newly constructed ceramic walls. Sulphates originate from impurities present in the raw materials used in construction ceramics production. Additionally, cement mortar with admixtures, used to integrate ceramics with concrete, serves as a source of carbonates [18,19,20]. It is worth noting that air pollution can also contribute to sulphate presence, as evidenced by research findings on efflorescence in historical buildings dating back to the nineteenth and twentieth centuries [21]. In older ceramic walls affected by efflorescence, various other groups of soluble salts, including chlorides and nitrates, are often found [22,23,24,25,26]. These salts are typically drawn into the material through capillary action from the surrounding soil. Another distinctive category of structures includes coastal facilities exposed to marine aerosols, which can lead to the formation of sulphate salts, such as arcanite: arcanite—K_2_SO_4_, thenardite—Na_2_SO_4_, hexahydryte—MgSO_4_·6H_2_O, epsomite—MgSO_4_·7H_2_O; and mixed-sulphate salts such as glauberite—CaNa_2_(SO_4_)_2_, aphthitalite—K_3_Na(SO_4_)_2_), bloedite—Na_2_Mg(SO_4_)2·4H_2_O), langbeinite—(K_2_Mg_2_(SO_4_)_3_, eugsterite—Na_4_Ca(SO_4_)_3_·2H_2_O, syngenite—K_2_Ca(SO_4_)·H_2_O, and polyhalite—K_2_Ca_2_Mg(SO_4_)_4_·2H_2_O [27]. It is well established that sulphate salts exert the most detrimental impact on ceramic masonry. Within this group, the most frequently encountered salts include potassium, sodium, magnesium, calcium, aluminum, and double sulphates. Notably, sodium and magnesium sulphates are the most deleterious [28]. The destructive potential of these salts is not only a result of their high solubility, which is highly temperature dependent (as illustrated in Figure 2), but also due to their capacity to form multiple hydrates, some of which are stable. The temperatures shown in Figure 2 can occur in ceramic brick walls.

For instance, according to Lindström [24], the crystallization of certain hydrated forms has the most severe destructive impact, including mirabilite (Na_2_SO_4_∙10H_2_O), epsomite (MgSO_4_∙7H_2_O), hexahydrate (MgSO_4_∙6H_2_O), kieserite (MgSO_4_∙H_2_O), and the anhydrous form of sodium sulphate, thenardite [24], in saturated solutions. E. Ruiz-Agudo et al. [30] investigated the corrosive effect of sulphate salts (sodium sulphate and magnesium sulphate) on porous limestone. They concluded i.a. that the crystallization of sodium sulphate causes subsequent stone layers to separate, whereas magnesium sulfate causes the formation and propagation of cracks. Differences in the form of damage caused by sulfate salt corrosion result mainly from different patterns of their crystallization. T. Chekai et al. [31] examined the corrosive effect of i.a. sodium sulphate on antique Dutch tiles. Based on the research results, they concluded that the harmful effect of sodium sulphate was greatest during the saturation/drying cycles, because of thenardite partial dissolution and mirabilite crystallization. Cracks appeared at the interface between ceramics and glaze, close to the areas with the highest evaporation which caused the material detachment. M. Koniorczyk and P. Konca [32] conducted research on the crystallization of sodium sulphate in porous building materials but also created a mathematical model of salt and moisture transport, considering the kinetics of the salt phase transformation. Calculations based on the mentioned model indicated that, the lower power in the rate law caused faster crystal growth of salt with smaller crystallization pressure. The supersaturation degree of the solution decreased with the faster growth of salt crystals, which was the impulse for crystallization pressure.

While much of the research has historically focused on the effects of individual sulphate salts, real-world systems frequently involve mixtures of multiple sulphate salts. Numerous double sulphate salts have been identified in various studies of building structures, including syngenite (K_2_Ca(SO_4_)_2_) [1,22,28], glaserite (K_2_Ca(SO_4)2_), bloedite (Na_2_SO_4_·MgSO_4_·4H_2_O), konyaite (Na_2_SO_4_·MgSO_4_·5H_2_O), eugsterite (2Na_2_SO_4_·CaSO_4_·2H_2_O) [1,22,33] and glauberite [25,34].

Although double salts are generally less harmful to masonry materials than single salts, many researchers consider it crucial to understand the conditions under which double sulphate salts form and their influence on materials [23,35,36]. This article demonstrates that under specific conditions, mixed solutions of sulphate salts can be more damaging to ceramic structures than individual sulphate salts. The objective of this study is to simulate the conditions when double salts become particularly harmful to the durability of porous ceramic products and to understand the corrosion mechanisms causing this type of destruction.

## 2. Materials and Methods

The primary phase of this research involved an experiment in which we recorded changes in the parameters of ceramic materials while subjecting them to cyclic immersion in corrosive solutions followed by drying. Before conducting this experiment, it was essential to prepare a suitable quantity of ceramic materials characterized by a porous structure, such as low-density masonry elements. Due to their high open porosity, these materials exhibit increased susceptibility to the detrimental effects of efflorescence. They were made of:

- Clay (10 vol.%) from the Oleśnica deposit (Świętokrzyskie Voivodeship, Poland), formed in Miocene in marine conditions (therefore it contains many carbonates), used in one of the largest wall ceramics factories in Europe, Wienerberger Oleśnica,
- quartz sand (10 vol.%), introduced to reduce the plasticity of the clay raw material,
- sawdust (15 vol.%), which creased the porosity of the fired material.

The chemical composition of raw clay was conducted using the PANalytical Axios mAX 4 kW WD XRF fluorescence spectrometer (Almelo, The Netherlands), equipped with a Rhodium source. The mineral composition of raw clay was analyzed using X-ray diffraction (XRD) phase composition tests. It was carried out using a PANalytical X’Pert Pro MD diffractometer (Amsterdam, The Netherlands), employing a Cu Ka1 X-ray line with the Bragg-Brentano standard setup that incorporated a Ge (111) Johansson monochromator for incident beam conditioning. The scanning range covered 10° to 70° with a step size and a measurement time of 20 s for each step.

The raw materials were added in a dry state. Consequently, the clay raw material was dried until reaching a constant weight and subsequently crushed to achieve a grain size smaller than 2 mm using a mechanical disintegrator. Following the mixing of raw materials, water was added until a plastic mass was attained. Using a laboratory vacuum screw press (Verdes), cuboid-shaped samples with dimensions 30 mm (width) × 20 mm (depth) × 150 mm (height) were molded from the mass. Upon drying, these samples were subjected to firing in a laboratory chamber furnace under the following conditions: temperature increased at a rate of 100 °C/h, held at 600 °C for 1 h, held at the maximum temperature of 950 °C for 2 h, followed by inertial cooling.

The basic parameters of the materials (water absorption, open porosity, flexural strength, compressive strength, and bulk density after sintering) were determined with following the methodology described in [37]. Water absorption was determined by comparing the mass of water absorbed by the sample after 48 h of saturation to the mass of the dry sample and was calculated using the formula:

(1) $$W_{a}\;=\;\frac{m_{s}-m_{d}}{m_{d}}\cdot 100\%$$

Open porosity was determined using the Archimedes method (ASTM-C373). Samples were weighed after 48 h of saturation and the following equation was used for the calculation:

(2) $$P=\frac{m_{s}-md}{m_{s}-m_{h}}\cdot 100\%$$

The three-point bending flexural test measures were conducted using a universal programmable test machine Cometech QC-508 (Taichung, Taiwan) with a press arm speed of 2 mm/min. The average characteristic dimensions were: sample height (*h*) = 18 mm, sample width (*b*) = 27 mm, and the span length (the distance between the two bottom roller support, (*S*) = 100 mm. The flexural strength was obtained using the formula:

(3) $$\sigma =\frac{3F_{F}\cdot S}{2b\cdot h^{2}}$$

Compressive strength tests were conducted on samples after the flexural strength test. Each half of the samples was rotated so that the compressed surface had an average dimension of 18 mm (width of research area = height of sample = *h*) × 25 mm (length of the press plate = length = l), and the sample height was approximately 27 mm. The tests were assessed using a universal test machine Cometech QC-508 (Taichung, Taiwan) by applying a loading rate of 0.05 N/mm^2^/s recording the breaking force during compression (*F_c_*), and calculating the strength according to the appropriate formula:

(4) $$C_{s}=\frac{F_{c}}{h\cdot l}$$

Bulk density of samples after sintering was calculated by dividing the material’s weight by its volume (determined by multiplying the measured dimensions) as in the formula:

(5) $$\rho =\frac{m_{d}}{v}$$

These determinations were conducted on six samples, and thus the results represent average values, along with their corresponding estimated standard uncertainties. A significant level of α = 0.05 was employed for the values derived from the Student’s *t*-distribution. The subsequent stage of the experiment involved the preparation of three corrosive solutions, detailed in Table 1.

The highest concentration of corrosive solution for red clay brick recorded so far is 25% and 30% [38]. However, it should be clearly noted that high concentrations, were used at low temperatures of −20 °C to 40 °C. In the experiment, highly concentrated solutions, close to supersaturation in 20 °C, were deliberately used so that salt crystallization would occur quickly, and that the concentration of corrosive salts would be high. The limiting solution was Na_2_SO_4_, whose solubility at room temperature of 20 ± 2 °C slightly exceeds the concentration of 15%. In real conditions, salt concentrations are much lower; however, during the drying of the product, supersaturation of solutions in the capillary pore spaces always occurs.

The corrosion cycles involved immersing the samples in solutions for 2 h and subsequently drying them at 105 °C for 22 h. The time of immersion was determined based on previous capillary action tests which showed that 2 h was sufficient to completely fill the capillary pores with water, hence a similar time was determined for corrosive solutions. The samples were weighed before the commencement of the cycles and after the conclusion of each cycle (following drying). Photographs were taken every 5 cycles. The determination of the fundamental material parameters was performed after completing 10 and 20 cycles, following the same procedures as ceramic materials before conditioning. Based on our own earlier research, we initially planned to carry out a maximum of 40 cycles; however, due to significant damage to some samples, further experiment was stopped after 20 cycles.

After completing 20 cycles, basic properties of ceramic materials, X-ray diffraction (XRD) phase composition tests, and microstructure examinations were conducted on the samples. The qualitative phase composition analysis of the sintered materials was carried out in the same conditions as described for the raw clay using.

For the microstructure and microchemical composition analysis of the sintered materials following the corrosion cycles, scanning electron microscopy was utilized in conjunction with the Energy-Dispersive X-ray Spectroscopy (EDS) method. This analysis was performed using the FEI Nova NanoSEM 200 (Eindhoven, The Netherlands), a scanning electron microscope provided by FEI Europe, equipped with an EDAX detector (Tilburg, The Netherlands). The experiments were carried out in low vacuums without steaming the samples with a conductive medium.

## 3. Results

### 3.1. Raw Materials

The chemical composition results for the clay raw material are provided in Table 2.

**Figure 3:**
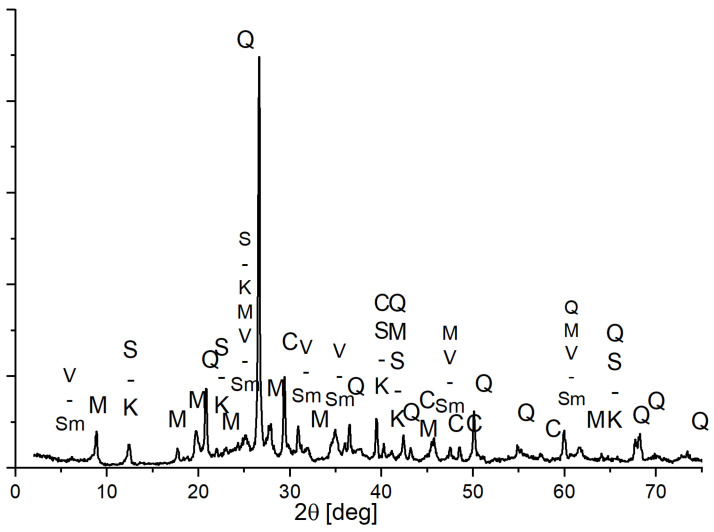
XRD pattern of raw clay from Oleśnica deposit, minerals: V-Sm—vermiculite-smectite minerals, I—mica and illite minerals, S-K—serpentinite-kaolinite minerals, Q—quartz, and C—calcite.

The Oleśnica clay raw material employed in this study exhibited a notable CaO content, exceeding 6.5%. This calcium oxide is primarily present in the form of dispersed calcite (whose presence was confirmed by XRD mineral composition tests—Figure 3) and it was indicated by the characteristic foaming observed upon exposure to 5% hydrochloric acid.

Oleśnica raw clay contains a relatively small amount of SO_3_ (Table 2), but even this amount may affect the equilibrium with corrosive solutions, reducing their solubility through the common ion effect. SO_3_ can occur as soluble or insoluble salts which, because of firing, pass into a soluble form. In the ceramic building materials industry, to prevent this effect of thermal changes, barium carbonate is added. It reacts with sulphates to form insoluble minerals. In this work, we did not introduce any additives reacting with soluble sulphates. In the phase composition of Oleśnica clay—Figure 3, sulphate phases are below the detection threshold by the XRD method.

Mineralogically, the clay used contains mixed-pack minerals of the vermiculite-smectite type, serpentinite-kaolinite minerals, and minerals from the mica/illite group, as well as non-plastic admixtures such as quartz and calcite (Figure 3).

### 3.2. Changes in Basic Physical Parameters of Ceramics

**Figure 4:**
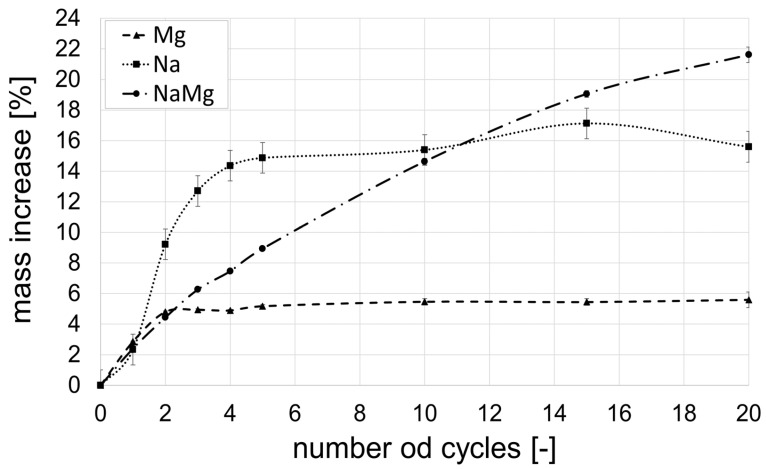
Damage to the volume of ceramic bricks caused by soluble salts.

Table 3 displays the fundamental parameters of the samples obtained before the commencement of the immersion cycles in solutions. As anticipated, it was feasible to produce samples with characteristics typical of porous ceramics. Figure 4 illustrates the percentage increases in the mass of the samples after exposure to corrosive solutions.

**Figure 5:**
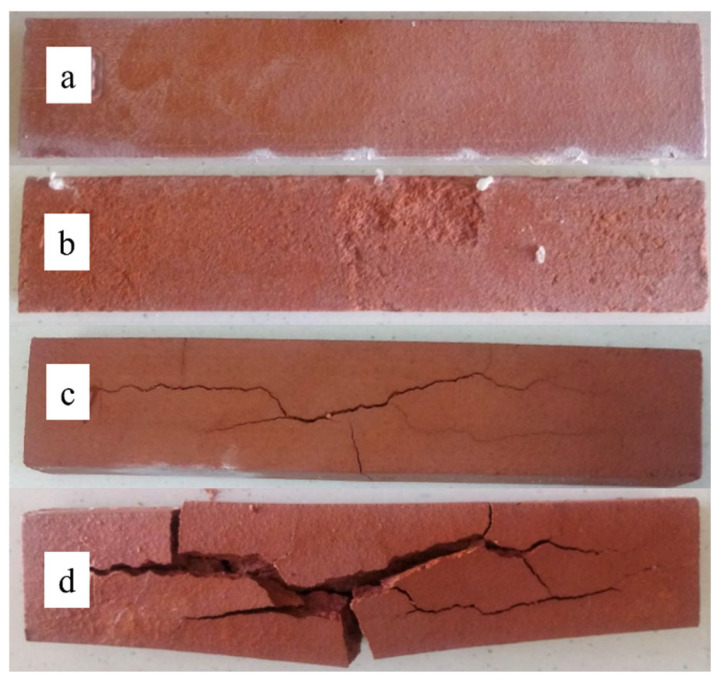
Sample after 20 conditioning cycles in (**a**) 15% MgSO_4_ solution, (**b**) 15% Na_2_SO_4_ solution, (**c**) 15 conditioning cycles in 7.5% Na_2_SO_4_ + 7.5% MgSO_4_ solution, and (**d**) 20 conditioning cycles in 7.5% Na_2_SO_4_ + 7.5% MgSO_4_ solution.

During the corrosive cycles, salt precipitates within the capillary pores of the samples. This phenomenon is most pronounced during the initial soaking and drying cycles. For cycles involving single salt solutions of Na and Mg, the conditioned material becomes sealed, and subsequent cycles result in negligible mass gains. There is a fundamental disparity in the quantity of salt deposited in the pores between the solutions. The smallest increase in mass, approximately 5%, resulting from salt deposition in the capillary pores, was observed in samples conditioned in a 15% magnesium sulphate solution. This is approximately 2.5 times less than that of a 15% sodium sulphate solution and 3 times less than that of a solution comprising a mixture of magnesium and sodium sulphate. The reason for these differences is probably the fundamentally different wettability of solutions in the ceramic material, which influences the capillary action forces. Table 4 provides a summary of the results, including the determination of the basic material parameters after 20 cycles of immersion in corrosive solutions and subsequent drying. Figure 5a–d display images of these samples.

During the immersion in corrosive solutions and subsequent drying of ceramic materials, a seemingly favorable sealing effect is observed. During the drying of samples soaked in a salt solution, crystallization of these salts occurs in the capillary pores of the product. During the process of soaking the product in the solution following drying, it is not possible to completely dissolve the crystallized salts in the capillary pores, because the solution of this salt is close to supersaturated and diffusion in the capillary pores is strongly limited. This leads to a reduction in the materials’ open porosity and water absorption, accompanied by an increase in their density, compressive strength, and bending strength. However, as reported in other studies by different authors, as the cycles progress, these trends begin to reverse, and the degradative impact resulting from the corrosive action of salt becomes predominant. The initial effects of this nature, such as surface exfoliation of the materials, are visible in Figure 5b.

What is most significant in the context of this research is the mass deterioration of materials exposed to the corrosive mixture of Na_2_SO_4_ and MgSO_4_ salts. The nature of the cracks observed in these materials differs from that observed with single salt solutions. As depicted in Figure 5c,d, the samples experience internal ruptures. The first cracks appear after 10 soaking–drying cycles. This case is remarkable, as similar instances are not widely documented in the available literature. Consequently, subsequent investigations primarily focused on unraveling the causes of this phenomenon.

### 3.3. Phase Composition of Materials Conditioned in Corrosive Solutions

**Figure 6:**
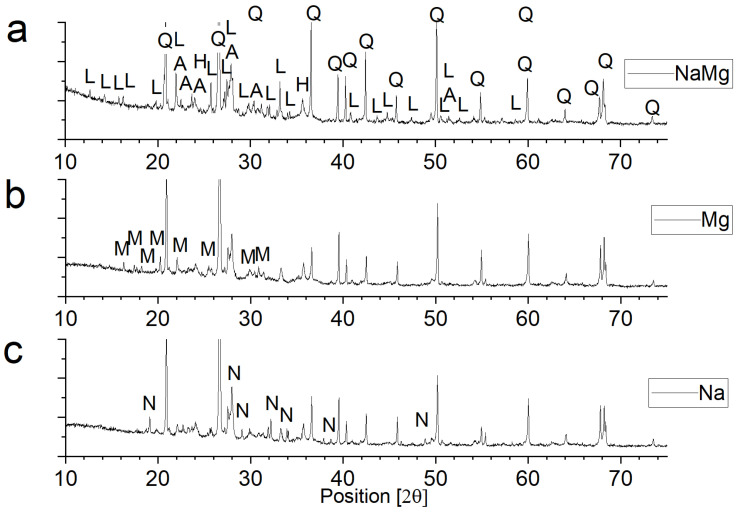
Phase composition of ceramics after 20 conditioning cycles in corrosive solutions: (**a**) 7.5% Na_2_SO_4_ + 7.5% MgSO_4_ solution, (**b**) 15% MgSO_4_ solution, and (**c**) 15% Na_2_SO_4_ solution. (Q—quartz: SiO_2_, A—albite (calcian): (Na,Ca)Al(Si,Al)_3_O_8_, H—hematite: Fe_2_O_3_, L—ploweite: Na_12_Mg_7_(SO_4_)_13_·15H_2_O, M—hexahydrate: MgSO_4_·6H_2_O, and N—thenardite: Na_2_SO_4_.).

Figure 6 presents the phase composition of samples following 20 cycles of immersion in corrosive solutions. The predominant phase component identified in all the samples was β-quartz, a low-temperature polymorphic form of quartz (card number: 01-086-1560). In addition to this, hematite (card number: 01-079-0007) and albite with low calcium content (card number: 01-076-0927) were consistently found in all samples. These phases are characteristic components of ceramics derived from clay materials.

The other phases detected in the samples result from exposure to corrosive solutions. In the case of samples conditioned in a 15% Na_2_SO_4_ solution, an additional phase observed is thenardite, an anhydrous sodium sulphate (card number: 01-086-0803). As for samples aged in a 15% MgSO_4_ solution, an additional phase present in the samples was the 6-water form of magnesium sulphate (card number: 00-024-0719).

In the case of the most damaged samples soaked in a salt mixture of Na_2_SO_4_ and MgSO_4_, an additional component was ploweite (according to [23] loeweite, card number: 00-029-1241), that is, 15-water double salt of sodium and magnesium sulphates with the total formula 6Na_2_SO_4_·7MgSO_4_·15H_2_O. The presence of this salt itself is not surprising. The available literature shows that it is one of the four stable double salts in the sodium sulphate and magnesium sulphate system—Figure 2. With the equilibrium content of these salts in solution, there is a high probability of the appearance of ploweite. However, this does not explain the observed phenomena, i.e., the gradual expansion inside the material. Ploweite, although it is a 15-water hydrate, does not show high hydration pressure. The mass fraction of crystallization water is less than 14%. For comparison, the percentage of crystallization water in mirabilite (Na_2_SO_4_·10H_2_O) is 56% and in epsomite (MgSO_4_·10H_2_O) is 51%.

### 3.4. Microstructure of Materials Conditioned in Corrosive Solutions

**Figure 7:**
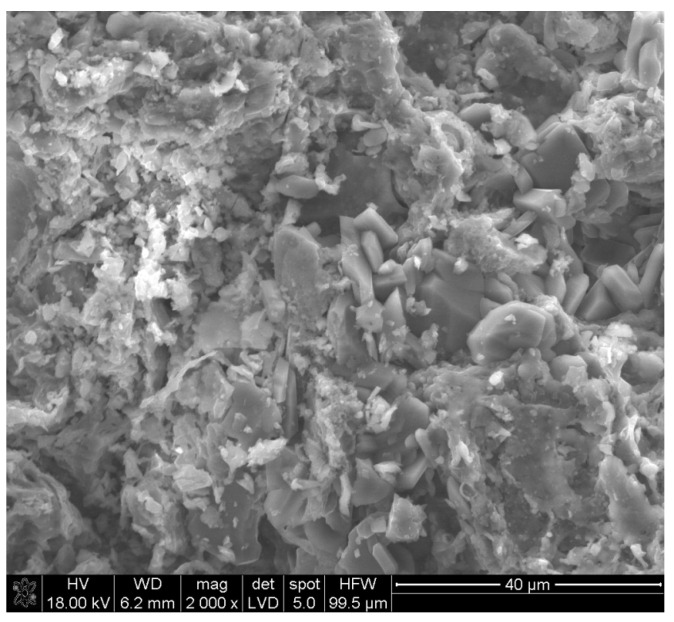
Microstructure of a ceramic sample conditioned in a mixture of sodium and magnesium sulphate salts.

The samples subjected to conditioning in a mixture of Na_2_SO_4_ and MgSO_4_ salt solution exhibited macroscopic damage. In this instance, SEM microscopic observations, accompanied by simultaneous microanalysis of specific points in the microstructure, aimed to identify the phases responsible for the damage. Figure 7 displays the microstructure of a sample conditioned in a mixture of Na_2_SO_4_ and MgSO_4_ salt solution at relatively low magnifications. This image presents the characteristic regular prismatic and cubic grains.

**Figure 8:**
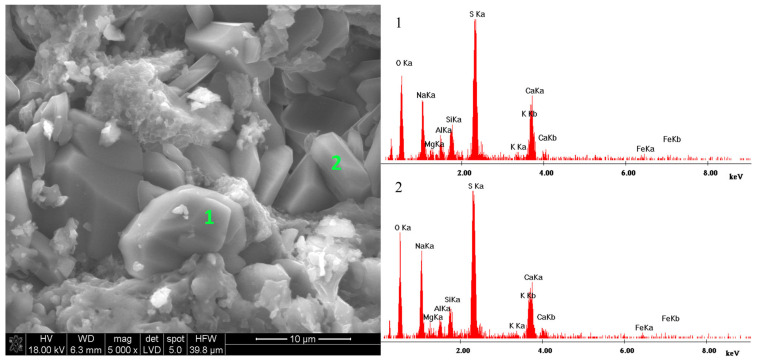
Microstructure of a ceramic sample conditioned in a mixture of sodium and magnesium sulphate salts; (**1**)—Microanalysis of point 1—glauberite: Na_2_SO_4_·CaSO_4_; (**2**)—Microanalysis of point 2—glauberite: Na_2_SO_4_·CaSO_4_.

Larger clusters are particularly visible in the upper right corner of Figure 7; therefore, this area was enlarged (Figure 8), and two EDS microanalyses were performed for the characteristic points (Figure 8(1,2)). Both microanalyses are practically identical. The dominant elements of these characteristic grains are S, O, Na, and Ca. The other recorded elements: Si, Al, and Mg are probably the background elements and not the observed phases themselves. The observed crystals are probably double salt; however, not ploweite but sodium and calcium sulphate. The EDS analysis does not provide precise information on whether it is glauberite Na_2_SO_4_·CaSO_4_ or Eugsterite 2Na_2_SO_4_·CaSO_4_·2H_2_O. However, grain morphology indicates the presence of two prismatic or tabular grains of glauberite in comparison to Eugsterite, the grains of which have a fibrous morphology.

**Figure 9:**
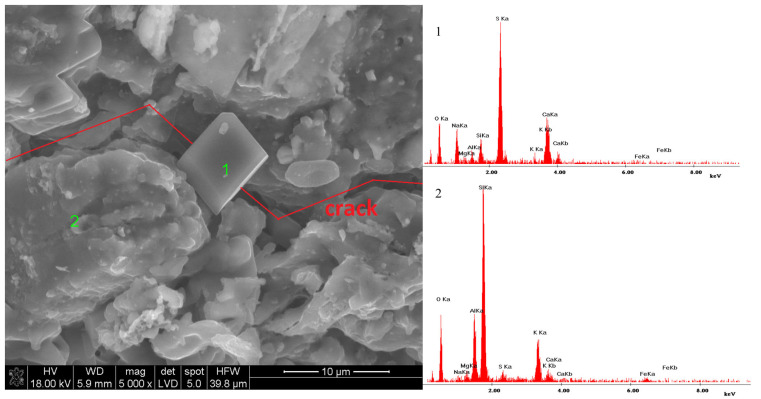
Microstructure of a ceramic sample conditioned in a mixture of sodium and magnesium sulphate salts—crack propagation; (**1**)—Microanalysis of point 1—glauberite: Na_2_SO_4_·CaSO_4_; (**2**)—Microanalysis of point 2—sintered clay mineral matrix.

Glauberite was frequently present in the sample and its formation could cause damage, as illustrated in Figure 9, which shows the axis of the cracks emerging from the grain. Figure 9(1) shows the analysis of this grain.

**Figure 10:**
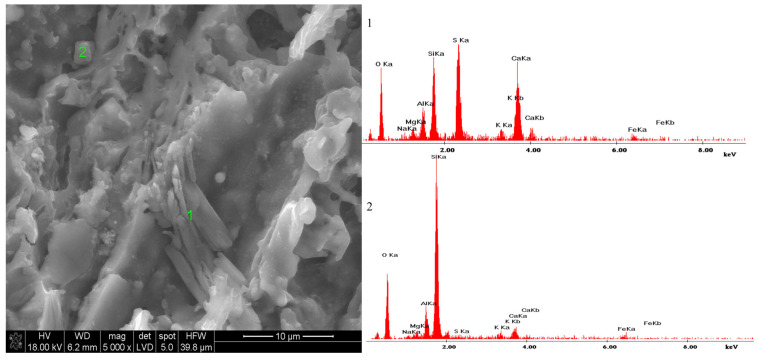
Microstructure of a ceramic sample conditioned in a solution of magnesium sulphate; (**1**)—Microanalysis of point 1—gypsum: CaSO_4_·2H_2_O on sintered clay mineral matrix; (**2**)—Microanalysis of point 2—quartz: SiO_2_.

Samples conditioned in individual solutions were also subjected to microscopic observations, which verified the presence of salts originating from the solutions in which the samples were conditioned. A noteworthy finding, as it later transpired, was the discovery of gypsum with an unusual microstructure in the sample immersed in sodium sulphate. To confirm this, Figure 10 displays an image of such a micro-area with a designated point, the microanalysis of which is presented in Figure 10(1).

In the sample conditioned in magnesium sulfate, calcium sulfate was identified—probably in gypsum form. Gypsum crystals were a recurring element of the microstructure of these samples. Gypsum usually forms large crystals, while those observed in the sample were small with an indistinct shape, superficially embedded on other elements of the microstructure. This was an important suggestion that gypsum grains crystallized from the aqueous solution during the drying of the samples. Interestingly, characteristic gypsum crystals were not observed in samples soaked in the Na_2_SO_4_ + MgSO_4_ salt mixture. This observation became the starting point for designing the model research presented in Section 3.5.

### 3.5. Model Research

SEM/EDS microscopic observations indicate that sodium and calcium sulphate is probably the phase that causes the damage of the ceramic materials. This phase is created because of the reaction of a corrosive solution (sodium sulphate) with the calcareous phases contained in ceramics. The presence of calcium-containing phases is the result of the use of clay-containing dispersed calcite. Therefore, the question arises of why the destructive phase appears in samples soaked in a salt mixture, and it cannot be detected in samples soaked in a sodium sulphate solution. To explain this phenomenon, a short model experiment was carried out.

The calcite contained in the clay raw material undergoes thermal dissociation during fire treatment of ceramics and free calcium oxide is formed.

**Figure 11:**
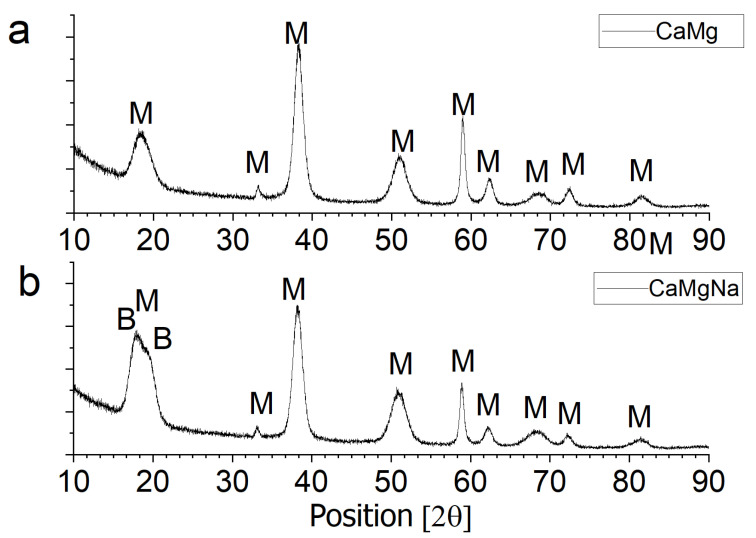
XRD pattern of sediments obtained in model tests: (**a**) precipitate formed in the reaction Ca(OH)_2_ solution and 15% MgSO_4_; (**b**) precipitate formed in the reaction Ca(OH)_2_ solution and 7.5% Na_2_SO_4_ + 7.5% MgSO_4_. B—bloedite: MgSO_4_·Na_2_SO_4_·4H_2_O; M—brucite: Mg(OH)_2_.

After being removed from the furnace, calcium oxide shows hygroscopic properties, because of which it is converted into calcium hydroxide (portlandite). Portlandite solubility is not high, which is 1.7 g per liter. However, when ceramics are soaked in corrosive solutions, this small presence of calcium ions in the solution may be of importance. To demonstrate this, three beakers were prepared to which a previously prepared saturated aqueous solution of calcium ions obtained by dissolving portlandite. It was added to the beaker in the volume of 100 mL. Then, one of the three solutions with compositions shown in Table 1 were added to each beaker in 5 mL volume proportion to Ca(OH)_2_ solution. A precipitate formed in the beakers in which the magnesium sulphate solution and the sodium and magnesium sulphate mixture were added. XRD analysis of this precipitate (Figure 11) indicated that it was magnesium hydroxide formed according to the reaction:

Mg^2+^ + SO_4_^2−^ + Ca^2+^ + 2OH^−^ = Mg(OH)_2_↓+ Ca^2+^ + SO_4_^2−^ (6)

In the process of drying such a solution, the second new phase that will appear after the solution is supersaturated will be calcium sulphate (possibly gypsum). This explains the presence of this phase, as shown in the SEM/EDS microstructure studies of samples soaked in the magnesium sulphate solution alone (Figure 10). The formed calcium sulphate is probably a precursor for the formation of the double salt of sodium and calcium sulphate. Therefore, this salt can only appear after conditioning in a salt mixture of sodium and magnesium sulphate.

The second phase recorded in the XRD study (Figure 11) was bloedite: MgSO_4_·Na_2_SO_4_·4H_2_O in the mixture of Ca(OH)_2_ and mixture of sodium and magnesium sulphate salts. The precipitation of bloedite is caused by the reduction in its solubility after the formation of Mg(OH)_2_, caused by the common ion effects SO_4_^2+^, which was present in excess.

Despite the slight supersaturation of all model research solutions with respect to gypsum, its presence was not observed in the XRD test results. It is likely that it is crystallized too little and occurs below the detection threshold of the XRD method.

## 4. Discussion

As mentioned in the introduction, the presence of salt in ceramic materials can cause massive damage, an example of which is presented in Figure 1. The mechanism of this type of damage is not fully understood, as it is difficult to simulate under laboratory conditions based on single corrosive salts. Studies managed to obtain this type of mass destruction by conditioning ceramic samples in a mixture of two solutions: sodium sulphate and magnesium sulphate. XRD analysis of ceramic samples, after conditioning, showed a significant content of ploweite, i.e., the hydrated double salt of the formula 6Na_2_SO_4_·7MgSO4·15H_2_O. The presence of this salt is not surprising. The phase is probably ubiquitous in the capillary pores of the material, but it is not necessarily directly responsible for the resulting damage. However, ploweite that resides in the capillaries may influence the shifting of the equilibrium states of aqueous salt solutions that remain in these capillaries during the conditioning of the samples. SEM microstructural observations of the surfaces formed during the damage showed the omnipresence of another double salt, sodium, and calcium sulphate. The morphology of the grains indicates that it may be glauberite. The crystallization of this phase probably directly causes expansion stress and destruction of the material. XRD studies do not confirm the presence of this phase, which is probably due to the partial coincidence of the peaks on the diffraction patterns with those of ploweite. Another explanation is the low stability of glauberite [39] attributed and possible degradation of this phase during the preparation of the sample for XRD studies. An important role in understanding the mechanism of glauberite formation was to determine the reason why glauberite did not form when ceramic samples were conditioned in a single sodium sulfate solution. Theoretically, all substrates for its formation are present there. As a result of the modeling study (Section 3.5), it was determined that the most likely necessary intermediate phase for the formation of glauberite is calcium sulfate, which can be formed because of the exchange reaction in solution of magnesium sulfate and solution of calcium hydroxide. This reaction takes place in the liquid phase and is solubility limited by the solubility of calcium hydroxide (1.7 g per liter). The source of calcium hydroxide is ceramics produced from clay raw materials containing calcite. Calcite dissociates thermally during the firing of ceramics, forming CaO. This oxide quickly hydrates in humid conditions, giving calcium hydroxide. variable humidity conditions (cycles) probably played a large role in the formation of glauberite, because only during the drying of the products was it possible for calcium sulphate to crystallize. While re-soaking with the salt mixture solution, favorable conditions occur for the formation of glauberite, because of the reaction of dissolved Na_2_SO_4_ with calcium sulphate grains.

Based on Figure 4, it can be inferred that the amount of glauberite gradually increased with each subsequent cycle, with the low solubility of calcium hydroxide in water serving as a limiting factor. The crystallization of glauberite led to the rapid development of microcracks in the sample, resulting in a continuous increase in the sample mass after each soaking–drying cycle.

Of course, this theory still needs to be confirmed by further research, as there are still many unknowns, e.g.,

- What role does the presence of ploweite play in the formation of glauberite, if any?
- What are the boundary conditions for the formation of the observed corrosion mechanism?

## 5. Conclusions

This article presents a simulation of volumetric damage in sintered porous ceramic materials (made of clay (75% vol.), quartz sand (10 vol.%), and sawdust (15 vol.%), fired at 950 °C) caused by the crystallization of double salts, specifically ploweite (6Na_2_SO_4_ 7MgSO_4_ 15H_2_O) and/or glauberite (CaSO_4_ Na_2_SO_4_). The fired ceramic samples were subjected to 20 cycles of soaking in corrosive solutions and drying. The following solutions were used: 15% MgSO_4_ solution, 15% Na_2_SO_4_ solution, and (7.5% Na_2_SO_4_ + 7.5% MgSO_4_) solution. The samples soaked in MgSO_4_ solution showed lower porosity and significantly higher compressive strength than the samples before corrosion tests. Samples soaked in Na_2_SO_4_ solution showed significantly lower porosity and slightly higher compressive strength than samples before corrosion tests. In both cases, single salts of magnesium or sodium sulfate were precipitated. The most aggressive solution was 7.5% Na_2_SO_4_ + 7.5% MgSO_4_, which caused internal cracking by crystallization of double salts ploweite (6Na_2_SO_4_ 7MgSO_4_ 15H_2_O) and/or glauberite (Na_2_SO_4_ CaSO_4_).

The destructive effect of single salts on porous ceramics has a completely different corrosion mechanism than the one that is the subject of this study. The only common denominator of these corrosions is the appearance of expansive forces related to the formation of crystals. In the case of single salts, this mechanism is usually of a surface nature, because it occurs during the drying of ceramic products. Then, an expansive process of crystallization of corrosive salts occurs, and sometimes during temperature changes also an expansive process of their hydration. In the case of the formation of glauberite, this process is completely different. Firstly, it probably occurs during the soaking of ceramic products and not during the drying itself, hence it does not necessarily occur on the surface of the products. In addition, the crystallization of glauberite is the effect of a direct chemical reaction, and this phase does not undergo hydration during thermal changes.

The research confirmed that the presence of dispersed calcite in the clay raw material used to produce ceramics may have a negative impact on the ceramic’s durability of the produced ceramics when exposed to mixtures of sulphate salts (MgSO_4_ and Na_2_SO_4_). The research has successfully simulated and provided an initial description of the corrosion mechanism within this system. The internal corrosion of sintered ceramics may be induced by the crystallization of soluble double salts, such as ploweite and/or glauberite. This marks a crucial starting point for further research, allowing us to identify the boundary conditions for the occurrence of the corrosion mechanism presented, and subsequently to devise strategies for its prevention.

## Figures and Tables

**Table 1:** Types of corrosive solutions.

Designation	Na	Mg	NaMg
Type and concentration	15% Na_2_SO_4_	15% MgSO_4_	7.5% Na_2_SO_4_ + 7.5% MgSO_4_

**Table 2:** Chemical composition of clay.

Component	SiO_2_	Al_2_O_3_	Fe_2_O_3_	CaO	MgO	K_2_O	Na_2_O	SO_3_	TiO_2_	Other	Loss on Ignition
Amount [wt%]	58.83	18.35	6.46	6.56	2.84	3.08	1.28	0.28	0.90	1.42	11.91

**Table 3:** Basic properties of ceramic materials before the experiment.

Properties	Water Absorption W_a_ [%]	Open Porosity P [%]	Compressive Strength C_s_ [MPa]	Flexural Strength σ [MPa]	Bulk Density After Sintering ρ [g/cm^3^]
Value	13.9 ± 0.2	24.4 ± 0.4	31.4 ± 1.2	8.6 ± 0.6	1.76 ± 0.05

**Table 4:** Basic properties of ceramic materials before the experiment (Mg—samples saturated with 15% MgSO_4_ solution, Na—samples saturated with 15% Na_2_SO_4_ solution, and NaMg—samples saturated with 7.5% MgSO_4_ and 7.5% Na_2_SO_4_ solution).

Properties	Water Absorption W_a_ [%]	Open Porosity P [%]	Compressive Strength C_s_ [MPa]	Flexural Strength σ [MPa]	Bulk Density After Sintering ρ [g/cm^3^]
Mg	10.4 ± 0.2	19.35 ± 0.3	44.8 ± 1.5	12.5 ± 0.8	1.85 ± 0.04
Na	2.47 ± 0.1	5.07 ± 0.2	32.7 ± 1.0	9.9 ± 0.8	2.03 ± 0.03
NaMg	break				

## Data Availability

The original contributions presented in this study are included in the article. Further inquiries can be directed to the corresponding author.

## Abbreviations

The following abbreviations are used in this manuscript:

$W_{a}$	Water absorption [%]
$m_{s}$	Mass of the sample saturated with water [g]
$m_{d}$	Mass of sample [g]
P	Open porosity [%]
$m_{h}$	Mass of the sample saturated with water in hydrostatic conditions [g]
σ	Flexural strength [MPa]
$F_{F}$	Maximum load force during flexural strength test [N]
S	Span length [mm]
b	Sample width [mm]
h	Sample height [mm]
$C_{s}$	Compressive strength [MPa]
$F_{c}$	Breaking force during compression [N]
l	Length of the press plate, length of the research area for compression strength [mm]
ρ	Bulk density [g/cm^3^]
v	Sample volume [dm^3^]
